# Optimization of Oxygen Pressure in HVOF Spraying for Enhanced Corrosion Resistance and Thermal Stability of Al-Cu-Fe Quasicrystalline Coatings

**DOI:** 10.3390/nano16130790

**Published:** 2026-06-23

**Authors:** Dilnoza Baltabayeva, Sherzod Kurbanbekov, Ali Coruh, Lyaila Bayatanova, Sattarbek Bekbayev, Berik Kaldar, Diyar Patchakhanov

**Affiliations:** 1 International School of Engineering, D. Serikbayev East Kazakhstan State Technical University, Ust-Kamenogorsk 070004, Kazakhstan; dilnoza.baltabayeva@ayu.edu.kz (D.B.); lbayatanova@edu.ektu.kz (L.B.); 2 The Research Institute “Natural Sciences, Nanotechnology and New Materials”, Khoja Akhmet Yassawi International Kazakh-Turkish University, Turkestan 161200, Kazakhstan; sattarbek.bekbayev@ayu.edu.kz (S.B.); berik.kaldar@ayu.edu.kz (B.K.); itsxxdi@gmail.com (D.P.); 3 Department of Physics, Faculty of Science, University of Sakarya, 54150 Sakarya, Turkey; coruh@sakarya.edu.tr

**Keywords:** corrosion, Al-Cu-Fe, HVOF, TGA, SEM

## Abstract

Al-Cu-Fe quasicrystalline coatings were deposited on AISI 321 stainless steel substrates by high-velocity oxy-fuel (HVOF) spraying at oxygen pressures of 3.0, 3.5, and 4.0 bar. The influence of oxygen pressure on the phase composition, microstructure, porosity, corrosion behavior, thermal stability, and microhardness of the coatings was investigated using X-ray diffraction (XRD), scanning electron microscopy coupled with energy-dispersive spectroscopy (SEM/EDS), ImageJ porosity analysis, electrochemical corrosion testing in 3.5 wt.% NaCl solution, simultaneous thermal analysis (TGA/DSC), and microhardness measurements. XRD analysis revealed the formation of quasicrystalline-related intermetallic phases together with Al, Fe_3_Al_13_, FeAl, Fe_3_O_4_, CuFe_2_O_4_, Cu_2_O, and CuO phases. The coating deposited at 3.5 bar exhibited the lowest porosity (5.37%), the most homogeneous microstructure, and the largest residual coating thickness after corrosion testing. SEM and EDS analyses indicated that corrosion preferentially initiated at pores, splat boundaries, and phase interfaces, while the coating produced at 3.5 bar demonstrated the most stable surface condition after exposure to a 3.5 wt.% NaCl solution. Thermal analysis showed that all coatings remained stable up to 900 °C. Sample (a) exhibited the lowest mass loss and the highest thermal stability, whereas sample (b) demonstrated the most favorable combination of structural integrity, phase ordering, coating density, corrosion-related performance, and thermal stability. Microhardness values of the coatings ranged from 754 to 778 HV, significantly exceeding that of the AISI 321 substrate. The results demonstrate that oxygen pressure is a critical parameter controlling the microstructure and functional properties of HVOF-sprayed Al-Cu-Fe coatings, with 3.5 bar providing the most balanced set of properties.

## 1. Introduction

Improving the corrosion resistance of structural materials operating in aggressive marine environments remains one of the major challenges in modern materials science. Protective coatings are widely employed to reduce corrosion damage and extend the service life of engineering components [1,2,3]. Among advanced protective materials, Al-Cu-Fe quasicrystalline coatings have attracted considerable attention due to their unique combination of high hardness, low friction coefficient, chemical stability, wear resistance, and corrosion resistance [4,5].

Various surface engineering techniques have been developed to improve the corrosion resistance of metallic structures operating in aggressive environments. Among these, conversion coatings such as zinc phosphating are widely used in the automotive industry due to their ability to improve coating adhesion and corrosion protection [6]. Recent studies have shown that low-temperature zinc phosphating, accelerated by triazole-based additives, can effectively enhance the corrosion resistance of microalloyed steels while simultaneously reducing processing energy consumption. Furthermore, alternative, environmentally friendly approaches have been proposed to mitigate corrosion processes in metallic materials exposed to aggressive environments [7]. For example, natural additives derived from spent coffee grounds have been shown to reduce the oxidative degradation of austenitic stainless steels exposed to soy biodiesel. These developments highlight the continuing need for advanced corrosion protection technologies and justify the research of new protective coatings with enhanced structural stability and corrosion resistance.

In parallel, thermally sprayed zinc duplex coatings have demonstrated outstanding long-term durability, maintaining effective corrosion protection of steel bridges for 40–50 years and potentially throughout their 100-year design life, although their performance strongly depends on coating quality and application conditions [8]. Furthermore, increasing industrial demands have stimulated the development of advanced zinc-based protective systems alloyed with elements such as Al, Mg, Sn, and Bi to improve corrosion resistance and mechanical performance [9]. Previous studies on HVOF/HVAF-sprayed Al-Cu-Fe quasicrystalline coatings have shown that deposition parameters significantly affect phase composition, oxidation behavior, porosity, and coating performance. However, the specific influence of oxygen pressure on the corrosion behavior of Al-Cu-Fe quasicrystalline coatings remains insufficiently understood. Therefore, the present study focuses on establishing the relationship between oxygen pressure during HVOF spraying, coating microstructure, and corrosion resistance.

Among quasicrystalline systems, particular attention is paid to Al-Cu-Fe alloys, in which an icosahedral quasicrystalline i-phase forms at standard cooling rates [10]. This phase is characterized by low density (4–5 g/cm^3^), high hardness (6–10 GPa), and a significant elastic modulus (70–100 GPa), which determines its potential suitability for protective and functional coatings [11]. However, studies of the corrosion behavior of cast Al-Cu-Fe alloys in salt solutions show that their corrosion resistance is determined to a greater extent by the phase and chemical composition of the alloy, rather than directly by the presence of the quasicrystalline phase [12,13].

Tests in 3–5% aqueous NaCl solutions indicate predominantly uniform dissolution of the Al-Cu-Fe alloy surfaces regardless of composition, with a decrease in corrosion rate observed as the proportion of copper-enriched phases increases. Potentiostatic studies revealed a tendency of these alloys toward pitting corrosion, accompanied by the formation of corrosion pits, the bottoms of which are covered by a porous layer of undissolved copper [14,15]. Similar results were obtained in climatic tests under salt fog conditions (5% NaCl, 35 °C), where no significant effect of the quasicrystalline phase content on the corrosion resistance of cast alloys was detected [16].

At the same time, it has been shown that the corrosion properties of Al-Cu-Fe alloys produced under non-equilibrium conditions differ significantly from those of cast materials [17]. This opens up opportunities to improve corrosion resistance by forming powder and film coatings using thermal spraying and gas-phase deposition methods [18,19]. Plasma spraying allows for the production of quasicrystalline coatings up to 1 mm thick; however, their corrosion resistance in aggressive media (H_2_SO_4_, NaOH) is in some cases lower than that of cast alloys, especially in alkaline solutions [20].

Thin-film coatings obtained by ion-plasma deposition methods, in particular magnetron sputtering, are considered more promising [21,22]. Such methods ensure high coating uniformity, low impurity levels, and structure formation at ultra-high cooling rates. It has been established that Al-Cu-Fe films up to 25 μm thick are generally amorphous and crystallize after vacuum annealing, whereas a crystalline structure forms when the thickness increases to ~130 μm [23,24,25,26]. The substrate temperature also has a significant effect on the phase composition: at temperatures above 400 °C, a predominantly quasicrystalline i-phase forms in the coatings [27].

Studies of the oxidation and tribological properties of Al-Cu-Fe quasicrystalline films have shown the formation of a passivating Al_2_O_3_ layer upon exposure to oxygen and water vapor, as well as high stability of the i-phase in aqueous and vapor environments [28,29,30]. The wear of such coatings in water is ceramic-like in nature and proceeds at a slower rate compared to a vapor environment.

The efficiency of ion-plasma deposition methods can be further enhanced by using an improved three-electrode ion-plasma sputtering technique, which allows the kinetic energy of the deposited atoms to be increased to 100–200 eV [31,32,33]. Under these conditions, the film structure forms during extremely non-equilibrium vapor-phase quenching, which significantly affects the phase composition and properties of the coatings [34]. It is known that an increase in the cooling rate leads to a decrease in the content of the quasicrystalline phase in the Al-Cu-Fe system [35]; therefore, doping with scandium is proposed to stabilize it [36].

It has been shown that the introduction of ~0.5 at.% Sc increases the fraction of the quasicrystalline i-phase by 20–25% and contributes to an increase in the corrosion resistance of Al-Cu-Fe film coatings in NaCl solutions of various concentrations [37].

Thus, the combination of out-of-equilibrium deposition and doping conditions appears to be a promising approach for creating corrosion-resistant quasicrystalline coatings to protect aerospace components operated in marine environments.

## 2. Materials and Methods

AISI 321 austenitic stainless steel conforming to AMS 5513 (supplied by Sandmeyer Steel Company, Philadelphia, PA, USA) was used as the substrate material (15 × 45 × 3 mm^3^), designed for machining and thermal spraying, was used as the substrate. This steel is alloyed with titanium, which increases its resistance to intergranular corrosion and thermal aging. Before coating application, the substrate was sandblasted using white corundum to increase surface roughness and improve coating adhesion (Table 1).

**Figure 1:**
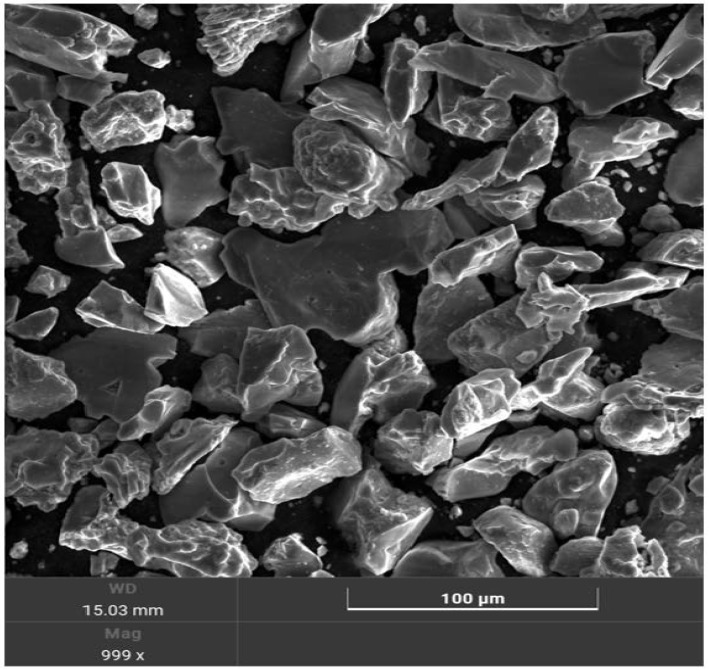
Powder particle size before coating deposition.

Aluminum (Al), copper (Cu), and iron (Fe) powders supplied by Hebei Suoyi New Material Technology Co., Ltd. (Handan, China), had a purity of greater than 99.5%. The surface morphology of the Al-Cu-Fe powders is shown in Figure 1. The particles exhibit an irregular polyhedral shape, featuring a combination of angular and plate-like forms-typical of materials obtained by mechanical alloying. This morphology generally results in lower flowability compared to spherical powders, but it provides a large surface area that is favorable for coating adhesion during thermal spraying.

The analysis of particle size showed a broad distribution, ranging from 10 to 80 μm. Specifically, the 10th percentile was 18 μm, the median (50th percentile) was 40 μm, and the 90th percentile was 75 μm. This particle size distribution is advantageous for smooth processing and consistent material delivery in the HVOF technique [9,10].

The coatings were deposited using a Termika-3 HVOF (A. Termika Ltd., Haifa/Nesher, Israel) spraying system. The HVOF process is characterized by a particle velocity of 500–700 m s^−1^ and a maximum particle temperature of approximately 1500 °C. Oxygen and propane were used as fuel gases, with oxygen pressure in the range of 0.8–1.0 MPa and propane pressure of 0.6–0.8 MPa. The oxygen and propane flow rates were 30–40 and 15–20 L min^−1^, respectively. Air cooling was applied during spraying with a cooling gas flow rate of 500–700 L min^−1^ at a pressure of 0.6 MPa. Al-Cu-Fe powder with a particle size fraction of −40 +15 μm was used as the feedstock material.

The spraying parameters were selected based on the operational characteristics of the HVOF process in [38] and the fundamental requirements for forming high-quality coatings. A spray distance of 250 mm between the nozzle and the substrate was chosen to ensure an optimal balance between particle temperature and impact velocity, thereby preventing excessive overheating and preserving the integrity of the quasicrystalline structure. A propane pressure of 1.9 bar was employed to maintain a stable combustion regime, enabling uniform heating of the powder particles without inducing complete melting. The oxygen pressure was varied in the range of 3.0–4.0 bar to optimize particle kinetic energy, promote coating densification, and enhance adhesive strength. An air pressure of 2.2 bar ensured complete fuel combustion, effectively reducing the risk of particle oxidation during flight toward the substrate (Table 2).

Maintaining the quasicrystalline structure of the coating was a key consideration throughout the process. The controlled spraying conditions ensured the preservation of the intrinsic properties of the Al_65_Cu_20_Fe_15_ alloy in the final coating. The interaction between particle characteristics, spraying parameters, and the resulting coating microstructure was systematically investigated, with particular emphasis on the role of oxygen pressure in controlling coating formation and performance.

The surface morphology and elemental composition of the coatings were examined using scanning electron microscopy (SEM) with a Tescan Vega 4 microscope (TESCAN ORSAY HOLDING, Brno, Czech Republic).

Prior to the corrosion tests, the coated samples were mechanically polished to obtain a uniform surface finish. The samples were subsequently cleaned and dried before electrochemical measurements. Corrosion tests were conducted using a P-20X potentiostat-galvanostat (Elins company, Chernogolovka, Moscow Region, Russia) in a step potentiostatic mode. The experiment consisted of 10 steps with a potential variation in the range of 1–15 mV. The step height was 1.5 mV with a dwell time of 30 s at each step. The total potential range was 2 V, the current range was up to 1500 mA, with a minimum detectable current of 200 nA. The data acquisition rate was 3.33 points/s. The tests were conducted in a 3.5% NaCl solution (pH 6.9–7.1) at room temperature. A two-electrode setup was used, where the test sample served as the working electrode, and the counter electrode ensured the electrical circuit was closed. The total duration of the experiment was 300 s.

Simultaneous thermal analysis (STA), including TGA and d DSC, was performed using an STA-200 instrument (Newgoer Testing Instruments Jinan, China). The study was performed under a nitrogen (N_2_) atmosphere to prevent oxidative processes. The sample was heated from room temperature to 900 °C at a rate of 5 °C/min. TGA and DSC were simultaneously recorded during the experiment. Thermogravimetric data were used to evaluate the thermal stability of the material and determine mass loss in a given temperature range. The DSC signal was analyzed to identify thermal effects (endo- and exothermic transitions) and calculate the corresponding enthalpy values using the instrument’s built-in software. The obtained curves were processed automatically, followed by the determination of the temperatures of the maximum thermal effects and the integral values of the thermal effects (ΔH, J/g).

## 3. Results

The selected parameters resulted in stable combustion, uniform spraying, and the formation of a high-quality coating on the AISI 321 stainless steel substrate. Owing to the optimal particle size and appropriate spray distance, the Al_65_Cu_20_Fe_15_ alloy powder remained in the flame for sufficient time to achieve effective heating and reached the substrate with adequate kinetic energy. This is confirmed by the uniform and homogeneous structure of the deposited layer.

The samples denoted as (a), (b), and (c) correspond to the coating conditions listed in Table 2. Such low porosity indicates effective particle deformation and limited oxide formation during deposition, which is typical for high-velocity oxy-fuel spraying under optimized processing conditions.

**Figure 2:**
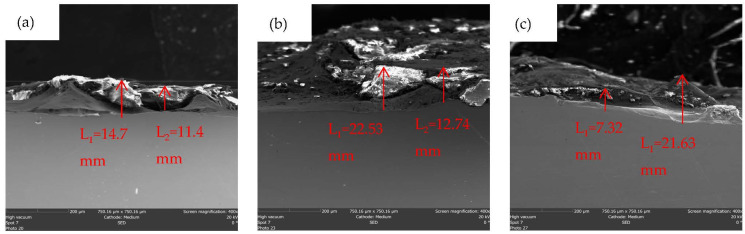
Micrographs of the thickness of Al-Cu-Fe coatings after corrosion: samples (**a**–**c**).

Before the corrosion experiments, a distinct interface was observed between the coating and the steel substrate. The coating thickness ranged from 40.87 to 115.41 µm. The measured thickness values before corrosion testing were 43.97 ± 3.0 µm for sample (a), 101.89 ± 13.5 µm for sample (b), and 92.56 ± 10.68 µm for sample (c). After corrosion testing, significant coating degradation was observed for all samples. Cross-sectional SEM analysis revealed that only a thin residual coating layer remained on the substrate surface. The residual coating thicknesses were approximately 13.0 5 ± 1.65 μm for sample (a), 31.5 ± 3.5 μm for sample (b), and 21.5 ± 8.13 μm for sample (c). Despite substantial thickness reduction after corrosion exposure, sample (b) retained the largest residual coating thickness, indicating superior corrosion resistance and structural integrity compared with samples (a) and (c). These results are consistent with the lower porosity and improved microstructural homogeneity observed for the coating deposited at an oxygen pressure of 3.5 bar (Figure 2).

**Figure 3:**
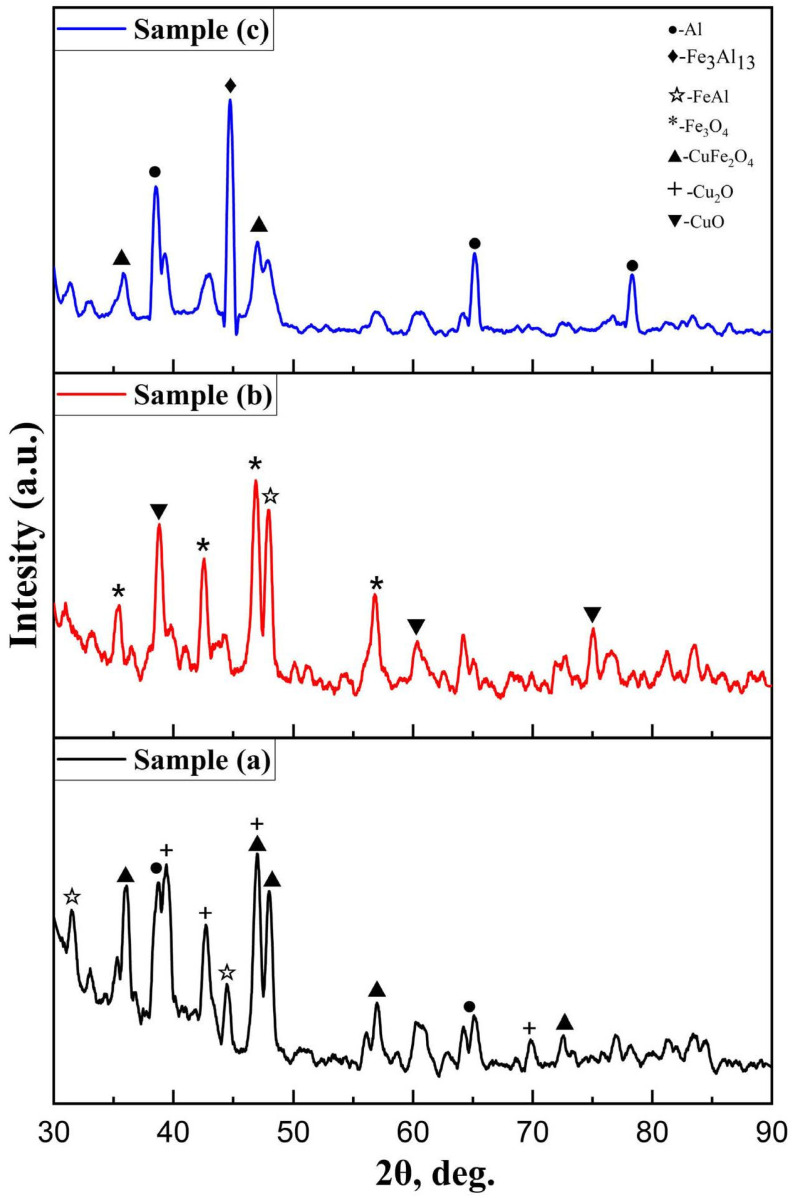
X-ray diffraction analysis.

X-ray diffraction analysis revealed that the phase composition of the HVOF-sprayed Al-Cu-Fe coatings depends strongly on the oxygen pressure applied during deposition. The diffraction patterns indicate the presence of several crystalline phases, including Al, Fe_3_Al_13_, FeAl, Fe_3_O_4_, CuFe_2_O_4_, Cu_2_O, and CuO. The coexistence of these intermetallic and oxide phases suggests the formation of a quasicrystal (Figure 3).

Among the investigated samples, the coating deposited at an oxygen pressure of 3.5 bar (sample b) exhibited the sharpest and most intense diffraction peaks, indicating enhanced crystallinity, improved phase ordering, and reduced structural defects. In contrast, samples deposited at lower (3.0 bar) and higher (4.0 bar) oxygen pressures showed broader and less intense reflections, suggesting increased microstructural heterogeneity and a greater influence of oxidation processes during spraying.

X-ray diffraction analysis results show that oxygen pressure may be related to structural relaxation and phase transformation processes in HVOF. The superior peak definition observed for sample (b) is consistent with its lower porosity, more homogeneous microstructure, and improved corrosion performance, indicating that an oxygen pressure of 3.5 bar provides the most favorable conditions for the formation of a dense and structurally stable Al-Cu-Fe coating.

The observed phase constitution is consistent with previously reported data for HVOF- and HVAF-deposited Al-Cu-Fe coatings [39], where a dual-phase structure composed of the quasicrystalline i-phase and β-phase is typically formed under non-equilibrium solidification conditions.

SEM observations were carried out in both secondary electron (SE) and backscattered electron (BSE) modes. SE imaging was used to evaluate the surface morphology and microstructural features of the coatings after corrosion testing, whereas BSE imaging was employed to distinguish phases and corrosion products based on compositional contrast and to investigate the corrosion mechanisms.

**Figure 4:**
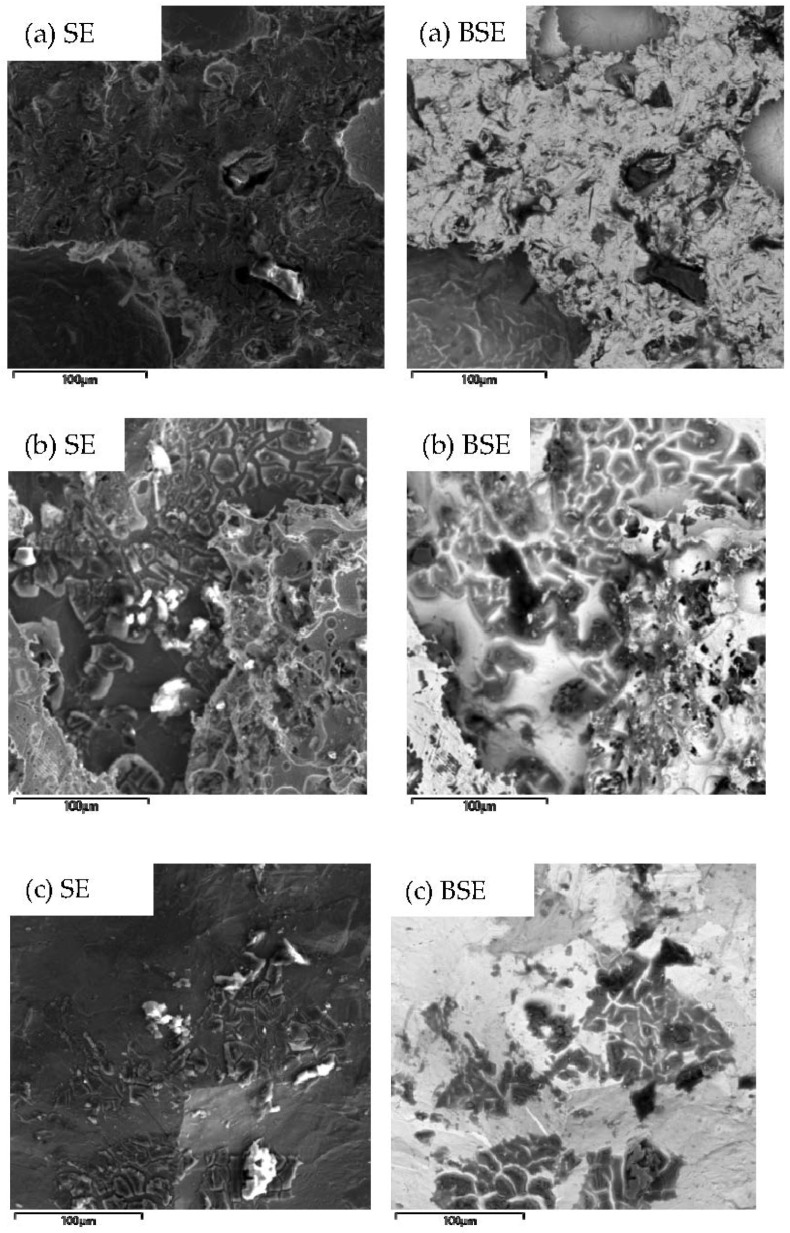
SEM image of the sample after corrosion in a 3.5% NaCl solution at room temperature.

Figure 4 shows SEM images of the coating surfaces following corrosion tests conducted in a 3.5% NaCl solution at room temperature SEM observations after exposure to 3.5% NaCl solution reveal localized corrosion attack characterized by micro-pitting, inter-splat degradation, and heterogeneous surface dissolution. Corrosion damage is preferentially initiated at coating defects, including pores, splat boundaries, and regions with compositional inhomogeneity.

Backscattered electron imaging indicates elemental redistribution during corrosion exposure, particularly enrichment and depletion of Cu-rich regions. This suggests the formation of micro-galvanic couples between intermetallic phases, which accelerates localized dissolution of the Al matrix.

The corrosion mechanism can therefore be described as a mixed process governed by:

(i) galvanic interactions between intermetallic constituents, and

(ii) chloride-induced breakdown of the passive Al_2_O_3_ layer.

These findings are consistent with previously reported corrosion behavior of Al-Cu-Fe-based coatings in chloride-containing environments, where preferential attack at phase boundaries is typically observed.

Al-Cu-Fe coating samples, which were produced under different oxygen pressures (3.0 bar; 3.5 bar; 4.0 bar), showed coating failure at potentials in the range of 1–15 mV; more precisely, as soon as a potential of 0 V is reached, we observe the appearance of numerous areas with corrosion defects of various shapes and sizes.

Thus, the Al-Cu-Fe quasicrystalline coating corrodes in aqueous sodium chloride solutions in accordance with an electrochemical mechanism. The rate of the cathodic process, accompanied by oxygen depolarization, is determined by the concentration of dissolved oxygen near the surface of the working electrode. Although sample (a) exhibited the lowest corrosion rate based on mass-loss measurements, sample (b) retained the largest residual coating thickness, exhibited the lowest porosity, and showed superior microstructural integrity after corrosion exposure. For comparison, in [40], the corrosion resistance of an Al-Cu-Fe alloy and its coating was studied in an oxygen-rich environment in a 5% NaCl solution under various time regimes of 1, 3, 5, and 8 days, and the results showed that the Al-Cu-Fe coating is more resistant to corrosion than the alloy.

**Figure 5:**
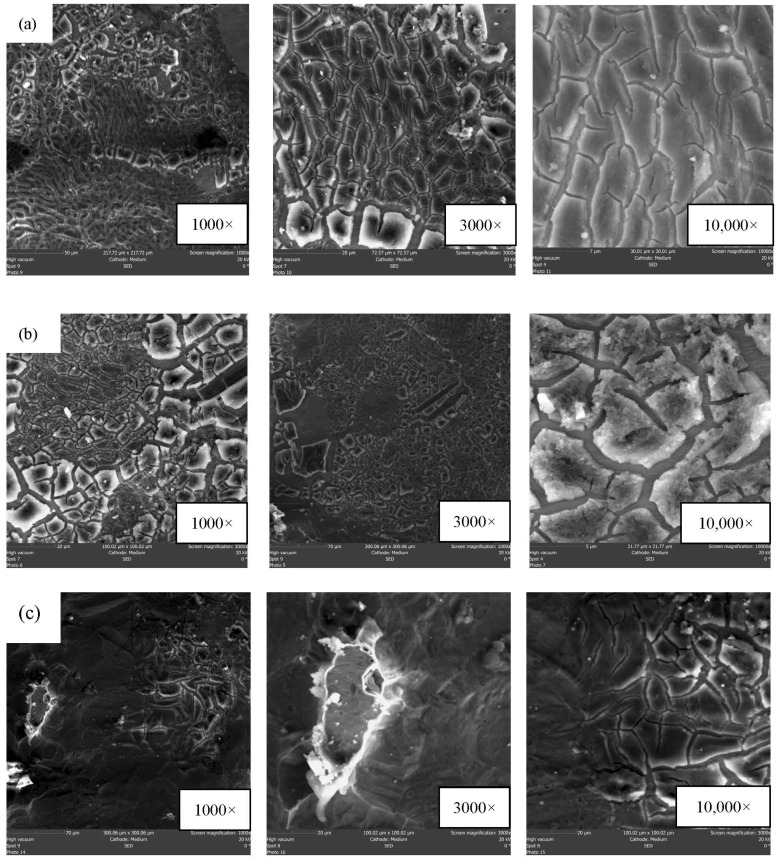
Surfaces of Al-Cu-Fe films after soaking for 300 s in a 3.5% aqueous NaCl solution at potentials ranging from 1 to 15 mV; magnifications of the samples after corrosion: 1000×; 3000×; 10000×.

Figure 5 shows SEM images at various magnifications taken after corrosion to provide more information on the condition of the coatings following electrochemical corrosion in a 3.5% NaCl solution. The image reveals corrosion defects of various shapes; the results obtained correlate with data from international studies, confirming general patterns and identifying specific features associated with variations in oxygen pressure during HVOF spraying. Wolf et al. (2020), [40] in their study of HVOF-deposited Al_62.5_Cu_25_Fe_12.5_ quasicrystalline coatings, demonstrated that such coatings exhibit a low coefficient of friction (≈0.1) and a specific wear rate of 1.7 × 10^−4^ mm^3^/N·m; however, in the presence of chlorides, corrosion resistance decreases, which is consistent with our data on the galvanic corrosion mechanism involving intermetallic phases. An important difference is that in Wolf’s work, the addition of chromium (Al_67_Cu_20_Fe_5_Cr_8_) improved corrosion resistance, whereas in our study, varying the oxygen pressure (3.0–4.0 bar) allowed us to control the structure without alloying [40].

Borisov et al. (2007) [41] investigated the corrosion resistance of thermally sprayed Al-Cu-Fe coatings containing a quasicrystalline phase and found that corrosion is localized and initiated at splat boundaries and around intermetallic inclusions. Our microstructural data at ×10,000 magnification, showing spherical clusters of supersaturated copper and needle-like Al(OH)_3_ crystals, fully confirm this mechanism. Furthermore, Borisov et al. (2007) and Borisova et al. (2006) demonstrated that the porosity of coatings and the degree of particle oxidation in flight critically influence corrosion behavior—a conclusion that we have quantitatively confirmed through electrochemical measurements in a corrosion failure at potentials 1–15 mV mode [41,42].

Feitosa et al. (2018) [43], while studying the effect of the oxygen-to-fuel (O/F) ratio on the structure of HVOF-deposited Al_59_Cu_25.5_Fe_12.5_B_3_ quasicrystalline coatings, showed that increasing O/F reduces porosity, but at excessively high values leads to material oxidation and the formation of undesirable phases. The authors determined that the optimal O/F ratio is slightly below 1.1 (the theoretical value for complete kerosene combustion). Our results at an oxygen pressure of 4.0 bar (Sample (c)) are consistent with this observation: maximum coating density is accompanied by signs of intermetallic oxidation, which reduces their cathodic activity and, paradoxically, increases corrosion resistance (minimum currents of 200–400 mA). At 3.0 bar (minimum pressure), increased porosity and maximum corrosion currents are observed, which is also consistent with the Feitosa model [43].

**Figure 6:**
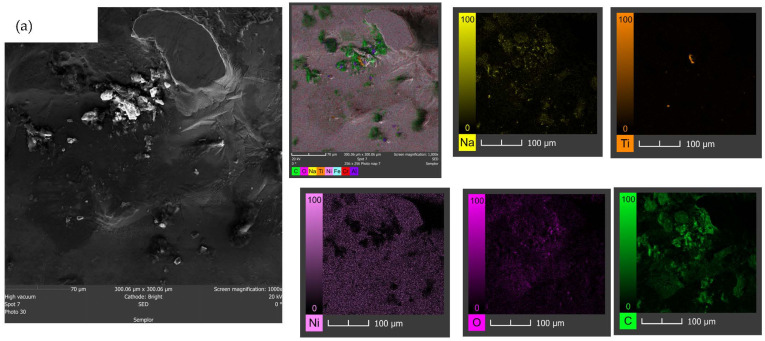

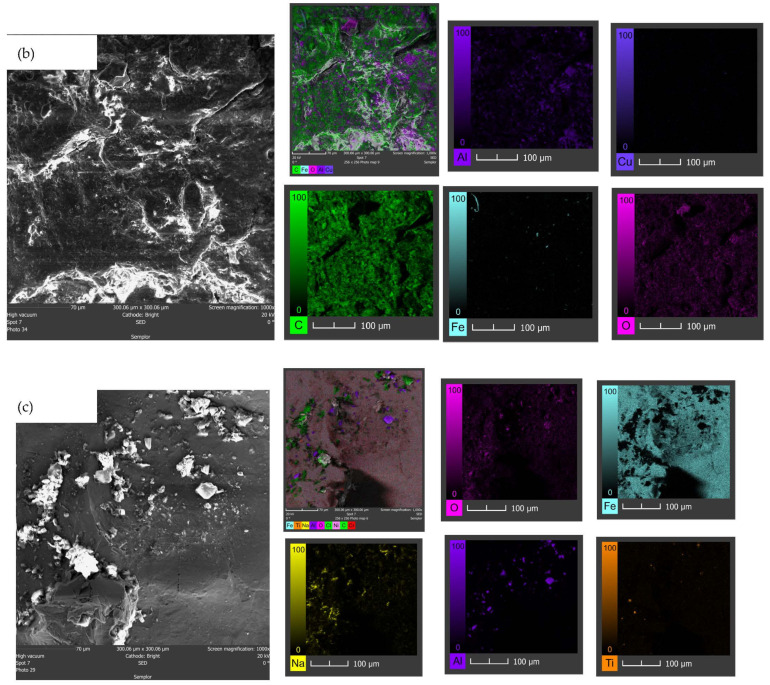
EDS Analysis after Corrosion Testing.

The EDS analysis performed after electrochemical corrosion testing revealed noticeable differences in surface composition among the investigated coatings. Among all samples, sample (b) exhibited the most stable surface condition after exposure to the corrosive environment (Figure 6), which is consistent with the electrochemical results.

Sample (a) showed the highest iron concentration (48.2 wt.%/22.1 at.%), indicating substantial exposure of substrate-related material after corrosion. Chromium and nickel remained detectable, while oxygen was present at 3.1 wt.%/4.9 at.%. Minor amounts of aluminum (0.8 wt.%/0.7 at.%), titanium (0.2 wt.%/0.1 at.%), and chlorine (0.9 wt.%/0.6 at.%) were also identified. The detection of chlorine confirms interaction of the coating surface with the chloride-containing electrolyte and suggests the development of localized corrosion processes.

For sample (b), the EDS spectrum was characterized by a high carbon content (71.0 wt.%/78.9 at.%) and the presence of oxygen (21.0 wt.%/17.5 at.%). Aluminum remained detectable (6.6 wt.%/3.3 at.%), whereas iron was observed only in a small amount (1.3 wt.%/0.3 at.%). Copper was not detected after corrosion testing. However, this apparent absence should be interpreted with caution, as it may result from surface oxidation, redistribution of copper within corrosion products, local compositional heterogeneity, or the limited sampling depth and detection capability of SEM–EDS analysis. Therefore, the present results do not allow a definitive conclusion regarding copper depletion from the coating. The observed surface composition is nevertheless consistent with the limited exposure of substrate-related elements after corrosion.

In contrast, sample (c) exhibited a considerably higher iron content (38.8 wt.%/16.0 at.%), together with chromium (10.5 wt.%/4.7 at.%) and nickel (5.8 wt.%/2.3 at.%). Oxygen was detected at 6.2 wt.%/8.9 at.%, reflecting the presence of oxide-containing corrosion products formed during exposure. Sodium was also detected (2.7 wt.%/2.7 at.%), confirming interaction with the corrosive electrolyte. The increased presence of substrate-related elements suggests more pronounced coating degradation and partial substrate exposure compared with sample (b).

Overall, the EDS results suggest that sample (b) experienced less surface degradation after corrosion testing compared with the other coatings. The observed surface composition is in agreement with the electrochemical results and microstructural observations, supporting the conclusion that sample (b) exhibited superior corrosion performance.

**Figure 7:**
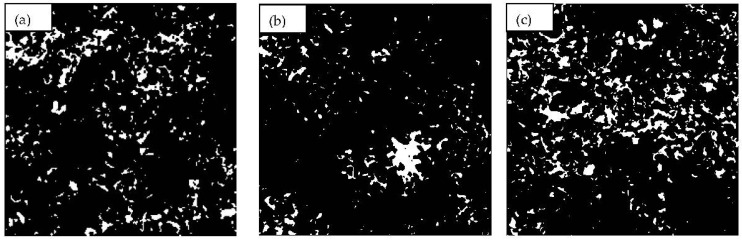
Porosity analysis of HVOF-sprayed Al-Cu-Fe quasicrystalline coatings using ImageJ software based on SEM surface liner micrographs: sample (**a**), sample (**b**), and sample (**c**) White regions correspond to pores identified during image processing.

The porosity of the coatings before corrosion testing was determined using ImageJ (1.52t version) software based on the analysis of surface micrographs obtained using SEM. The measured porosity values were 8.61% for sample (a), 5.37% for sample (b), and 11.02% for sample (c) (Figure 7). The average porosity of the investigated coatings was 8.33 ± 2.83%. Among the studied samples, sample (b), deposited at an oxygen pressure of 3.5 bar, exhibited the lowest porosity, indicating the formation of a denser and more homogeneous microstructure. In contrast, samples (a) and (c) showed higher porosity levels, which may negatively affect the barrier properties and corrosion resistance of the coatings. These results suggest that an oxygen pressure of 3.5 bar is optimal for the deposition of Al-Cu-Fe quasicrystalline coatings under the investigated HVOF spraying conditions, as it promotes the formation of coatings with minimal porosity and improved structural integrity.

Wan Peng et al. (2023) [44] investigated the effect of heat treatment on the properties of Al-Cu-Fe HVOF coatings and showed that after spraying, the content of the quasicrystalline I phase is ≈78.7%, and that of the β phase is ≈21.3%; after heat treatment at 550–600 °C, the β-phase disappears and the proportion of the I-phase increases, accompanied by a rise in hardness to 674 HV and improved corrosion resistance in 3.5% NaCl. The authors also found that sealing treatment (pore sealing) further enhances the protective properties. The results suggest that optimization of oxygen pressure may provide some microstructural benefits similar to those reported after heat treatment. The 3.5 bar mode yields an optimal combination of density and phase composition, close to the results of heat treatment at 600 °C [44].

Ryabtsev et al. (2019, 2020) [45] studied the corrosion properties of thin Al-Cu-Fe films produced by ion plasma spraying and demonstrated that the addition of 0.5 at.% scandium enhances corrosion resistance in NaCl solutions. They also established that in cast Al-Cu-Fe alloys, alongside the icosahedral I phase, the crystalline phases λ-Al_13_Fe_4_, β-AlFe(Cu), τ-AlCu(Fe), and θ-Al_2_Cu, with the film coatings exhibiting higher corrosion resistance due to their nanodispersed structure (grain size ≈3 nm) [18,45].

Our HVOF coatings occupy an intermediate position between coarse-grained cast alloys and nanostructured films. This explains why at 4.0 bar (Sample (c)) (minimum porosity, but some oxidation) the corrosion currents are higher than those of nanostructured films (200–400 mA versus < 100 mA in Ryabtsev’s work).

The corrosion rate was estimated from the mass loss during electrochemical testing in 3.5% NaCl solution over 300 s. The initial substrate exhibited a mass-loss rate of 2.03·10^−4^ g/s, ndicating pronounced degradation in the chloride-containing electrolyte. In comparison, sample (a) demonstrated the lowest corrosion rate of 4.0·10^−5^ g/s, reflecting improved corrosion resistance of the coating. Samples (b) and (c) showed intermediate corrosion rates of 1.13·10^−4^ g/s and 1.2·10^−4^ g/s, respectively. The average corrosion rate for all investigated samples was (1.19 ± 0.67)·10^−4^ g/s, where the deviation reflects the variation in corrosion behavior between the substrate and coated samples.

According to the work by B. I. Wehner and U. Köster [46], during high-temperature oxidation of Al-Cu-Fe alloys in air, linear or parabolic oxidation kinetics are observed, accompanied by the formation of oxide layers and the growth of oxide nodules already in the early stages (within the first few hours at 800 °C).

A comparison with the data of A. A. Teplov et al. shows that in polymer composites containing Al-Cu-Fe quasicrystalline fillers, thermal stability depends significantly on the environment: oxidative degradation is observed in air (starting at ~200–240 °C), whereas in an inert atmosphere the materials are stable up to ~380 °C. In the present work, a similar trend is confirmed; however, the temperature range of stability is significantly expanded (up to 700–900 °C), which is explained by the absence of a polymer matrix and the higher thermal stability of the metallic quasicrystalline system [47,48].

Of particular interest is the fact that, despite complete mass stability, intense thermal effects are observed for the first sample. This is consistent with the notion that phase and structural transformations can occur in quasicrystalline alloys without a change in mass, in contrast to polymer systems, where thermal effects are directly linked to degradation.

**Figure 8:**
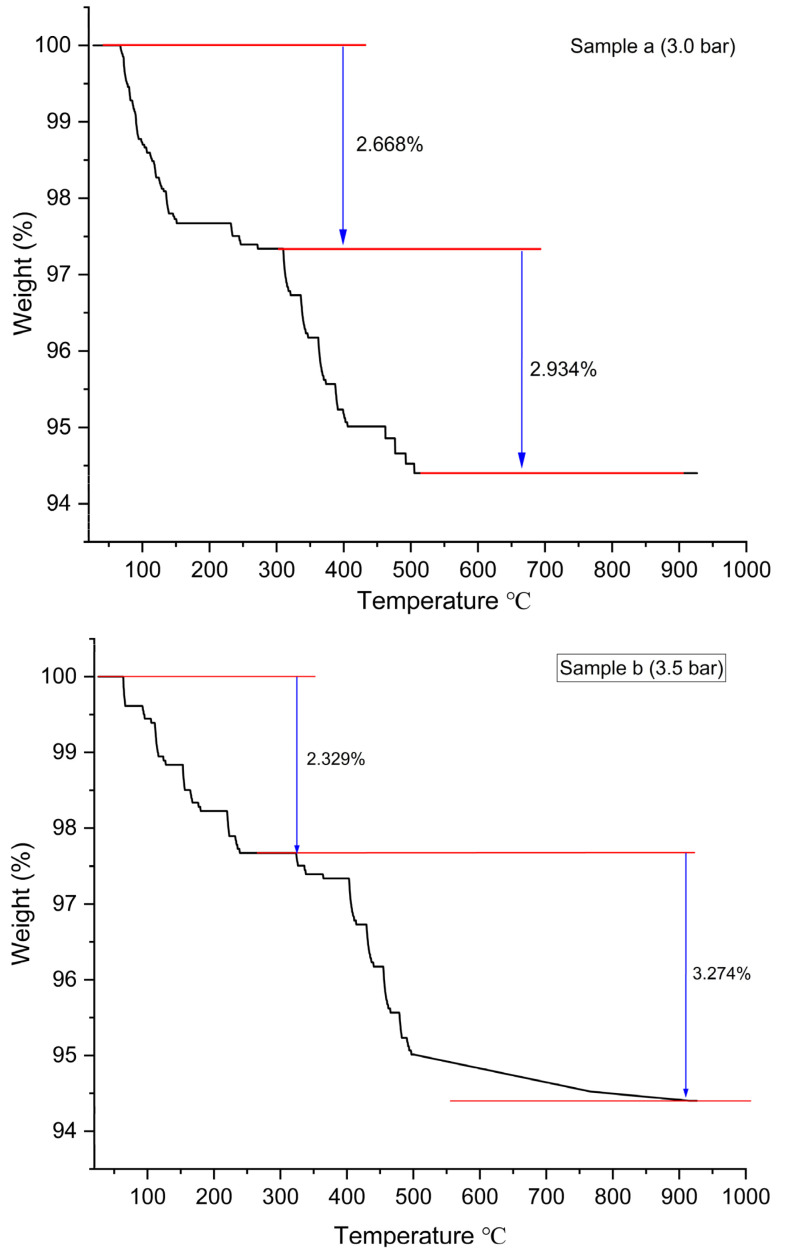

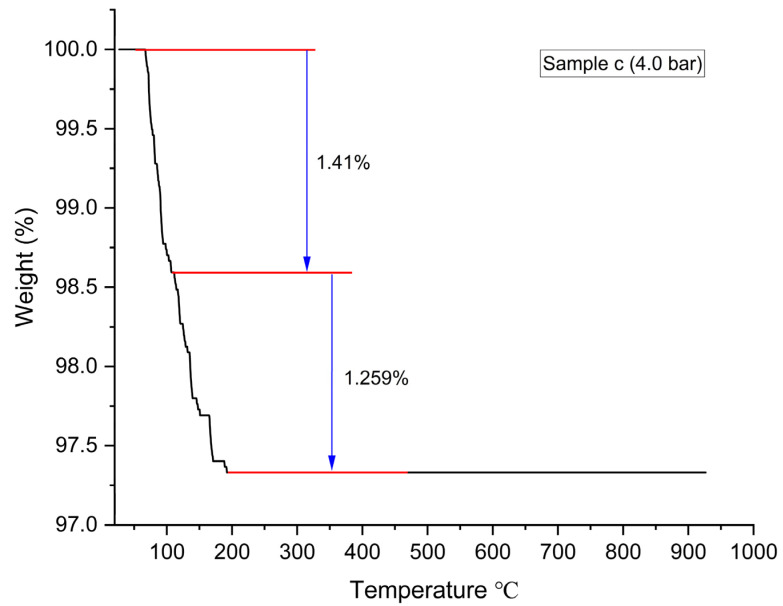
TGA curves for samples: (**a**–**c**).

TGA revealed significant differences in the thermal behavior of the three Al-Cu-Fe quasicrystalline coatings (Figure 8). Sample (a) exhibited the highest thermal stability among the investigated coatings. The TGA curve revealed two stages of mass loss: an initial loss of approximately 2.668%, attributed to the removal of adsorbed moisture and volatile species, followed by a second mass loss of approximately 2.934% associated with the release of more strongly bound components. After these stages, the mass remained essentially constant up to 900 °C, indicating the absence of significant decomposition processes. Simultaneously, the DSC curve showed pronounced thermal effects, suggesting that the observed changes were primarily related to structural relaxation and phase transformations within the quasicrystalline coating rather than thermal degradation. This behavior is characteristic of quasicrystalline alloys, where structural rearrangements may occur with minimal changes in mass.

Sample (b) (3.5 bar oxygen pressure) demonstrated intermediate thermal stability. An initial mass loss of approximately 2.329% occurred below 300–350 °C and was attributed to the removal of adsorbed moisture and volatile surface species. Further heating resulted in a gradual additional mass loss of about 3.274% up to 900 °C, which may be related to slow structural rearrangements and the release of more strongly bound components. Above 500 °C, the rate of mass loss decreased considerably, indicating relatively good thermal stability at elevated temperatures.

Sample (c) exhibited the lowest thermal stability among the investigated coatings. A mass loss of approximately 1.41% was observed in the temperature range of 59–274 °C, followed by continued mass reduction during further heating. The total mass change reached approximately 1.259% by 900 °C, suggesting the presence of a larger fraction of volatile species and possible partial decomposition processes. Compared with samples (a) and (b), sample (c) showed the greatest susceptibility to thermally induced degradation.

Overall, the thermal stability of the coatings decreased in the order: Sample (a) > Sample (b) > Sample (c). The results indicate that the coating produced under the conditions corresponding to sample (a) possesses the highest resistance to thermally activated structural and compositional changes, whereas sample (c) is the least stable at elevated temperatures.

**Figure 9:**
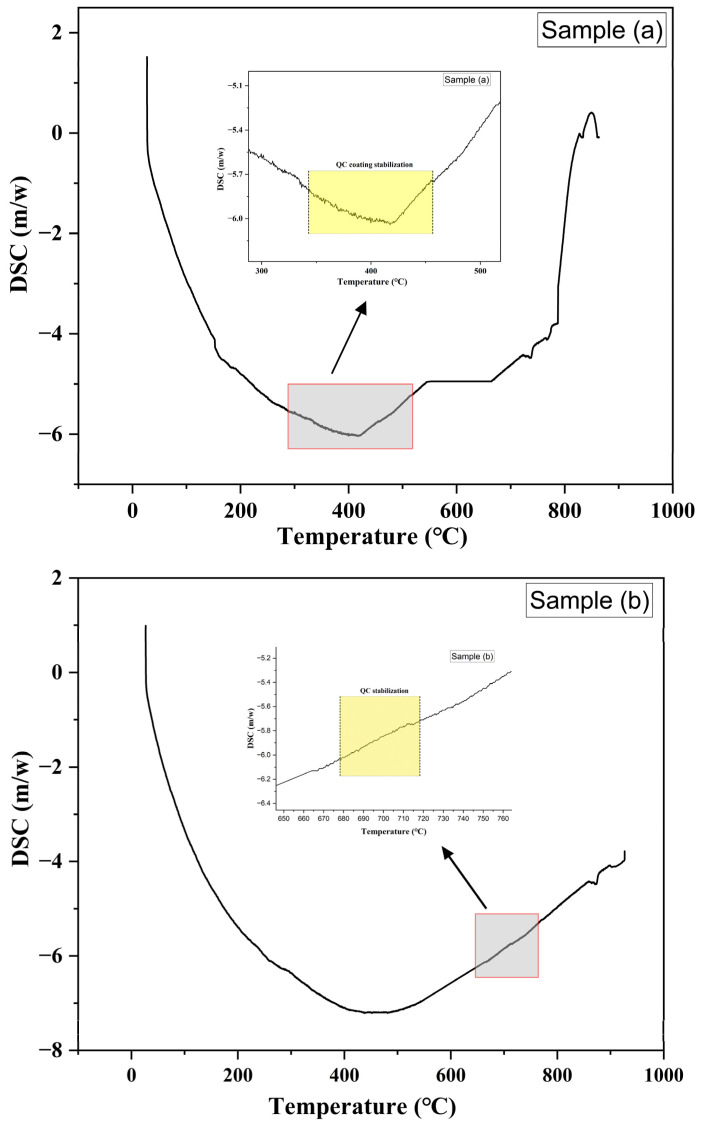

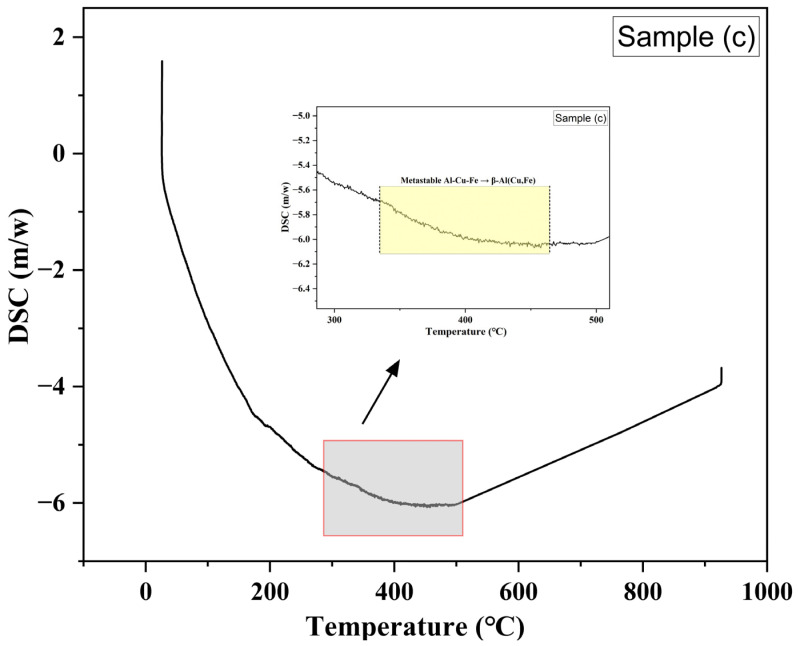
DSC curves of HVOF-sprayed Al-Cu-Fe quasicrystalline coatings deposited at different oxygen pressures.

Analysis of the DSC curves revealed significant differences in the thermal behavior of quasicrystalline Al-Cu-Fe coatings deposited at different oxygen pressures. Sample (a) demonstrated the highest thermal stability, as evidenced by the absence of significant mass loss over the entire temperature range studied [25]. However, a broad thermal effect was observed in the range from approximately 300 to 500 °C with a maximum at 410.3 °C and a corresponding enthalpy change of −16.31 J/g. This effect is explained by the relaxation of the thermally deposited metastable structure, a decrease in residual stresses, and solid-state structural rearrangements, including the may be associated with structural relaxation and phase rearrangement processes. The broad peak shape indicates that the process occurs gradually rather than through an abrupt phase transformation. At higher temperatures in the range of 650–725 °C, a weak thermal effect was observed, peaking at approximately 672.4 °C and possessing an enthalpy of approximately −0.006 J/g. This behavior is associated with further stabilization and ordering of the icosahedral quasicrystalline phase, accompanied by a gradual decrease in may be associated with structural relaxation and phase rearrangement processes (Figure 9).

Compared to sample (a), samples (b) and (c) exhibited more pronounced thermal instability, as evidenced by measurable mass loss upon heating. Sample (b), deposited at an oxygen pressure of 3.5 bar, exhibited moderate thermal stability, characterized by an initial mass loss due to desorption of physically adsorbed particles, followed by slow structural rearrangements. However, this sample demonstrated the lowest porosity (5.37%) and the most uniform microstructure among all the coatings studied, indicating that the selected spraying conditions contributed to the formation of a dense and structurally stable coating. In contrast, sample (c) exhibited the highest porosity (11.02%) and the lowest thermal stability, suggesting the presence of a higher number of structural defects, metastable regions, and weakly bonded particles. The increase in mass loss observed upon heating is consistent with a decrease in the structural integrity of this coating.

Overall, thermal analysis shows that oxygen pressure has a significant impact on the microstructural stability of quasicrystalline Al-Cu-Fe coatings. While sample (a) demonstrated the greatest resistance to thermally activated transformations, sample (b) combined good thermal stability with the lowest porosity and the most compact microstructure. Therefore, an oxygen pressure of 3.5 bar can be considered the optimal application condition, providing the best balance between coating density, structural homogeneity and thermal stability.

Although sample (a) demonstrated the lowest mass loss after corrosion testing, overall assessment of coating performance requires consideration of additional structural and functional characteristics. Mass loss reflects the total amount of material removed during corrosion but does not fully describe the integrity of the remaining coating or the mechanisms driving degradation.

Sample (b) demonstrated the lowest porosity, a more uniform microstructure, improved phase distribution, and the highest residual coating thickness after corrosion. These characteristics indicate a more effective barrier against electrolyte penetration and reduced susceptibility to localized degradation. Conversely, the lower mass loss observed for sample (a) may be due to differences in the mechanisms of formation and removal of corrosion products rather than superior coating protection.

Thus, the apparent discrepancy between gravimetric results and microstructural observations suggests that corrosion behavior is controlled not only by total mass loss but also by coating continuity, defect density, corrosion product stability, and substrate exposure. Taking into account all the characterization results, sample (b) demonstrated the most favorable combination of structural stability, thermal behavior and corrosion resistance among the coatings studied.

**Figure 10:**
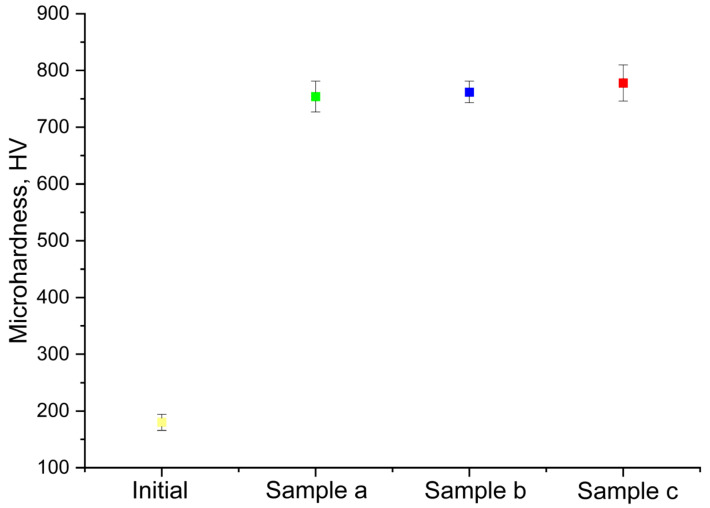
Average hardness values of the base steel and Al_65_Cu_20_Fe_15_ coatings at different oxygen flow rates.

Figure 10 presents the average microhardness values of Al_65_Cu_20_Fe_15_ coatings deposited via the HVOF method under varying oxygen flow rates. The coatings exhibited a significant enhancement in microhardness, increasing by a factor of 3.6–4.2 relative to the AISI 321 substrate. The substrate microhardness ranged from 180 to 210 HV, with an average of 195 HV and a standard deviation of ±10.2 HV, consistent with typical hardened AISI 321 steel. The average microhardness of the first, second, and third coatings was 754, 762, and 778 HV, respectively.

The overall mean microhardness of the coatings was 764.6 HV, with a standard deviation of ±10.0 HV, indicating a high degree of homogeneity among the tested samples.

## 4. Conclusions

Al-Cu-Fe quasicrystalline coatings were successfully deposited on AISI 321 stainless steel substrates by the HVOF process under different oxygen pressures. The influence of oxygen pressure on the phase composition, microstructure, porosity, corrosion behavior, thermal stability, and microhardness of the coatings was systematically investigated.

XRD analysis revealed the presence of quasicrystalline-related intermetallic structures together with Al-, Fe-, and Cu-containing phases. Oxygen pressure significantly affected phase development and coating quality during deposition. Porosity analysis performed using ImageJ showed values of 8.61%, 5.37%, and 11.02% for samples deposited at 3.0, 3.5, and 4.0 bar, respectively. The coating produced at 3.5 bar exhibited the lowest porosity and the most homogeneous microstructure.

Corrosion testing in 3.5 wt.% NaCl solution demonstrated that degradation preferentially initiated at pores, splat boundaries, and phase interfaces. Cross-sectional SEM observations showed substantial coating degradation after corrosion exposure; however, sample (b) retained the largest residual coating thickness and exhibited the most stable microstructural condition among the investigated coatings. SEM and EDS analyses further confirmed that sample (b) experienced the lowest degree of surface deterioration.

Thermal analysis indicated that all coatings remained stable up to 900 °C. Sample (a) showed the lowest mass loss during heating and therefore exhibited the highest thermal stability in terms of mass retention. At the same time, DSC results revealed structural relaxation and phase-ordering processes characteristic of Al-Cu-Fe quasicrystalline materials.

Microhardness measurements demonstrated a significant improvement in surface mechanical properties, with coating hardness values ranging from 754 to 778 HV, which were approximately four times higher than that of the AISI 321 substrate.

Considering all experimental results, sample (b), deposited at an oxygen pressure of 3.5 bar, exhibited the most favorable combination of low porosity, microstructural homogeneity, residual coating integrity after corrosion testing, good thermal stability, and enhanced mechanical performance. Although sample (a) demonstrated the highest thermal stability based on mass-loss measurements, the overall balance of structural, corrosion-related, thermal, and mechanical properties indicates that an oxygen pressure of 3.5 bar represents the optimal deposition condition for producing high-quality Al-Cu-Fe quasicrystalline coatings intended for operation in chloride-containing environments.

## Figures and Tables

**Table 1:** Chemical composition of AISI 321.

C	Mn	P	S	Si	Cr	Ni	Ti	Fe
0.08	2.0	0.045	0.03	1.0	17.0–19.0	9.0–12.0	0.5	Rest

**Table 2:** HVOF Conditions.

Propane (Bar)	Oxygen (Bar)	Air (Bar)	Distance (mm)
1.9	3.0	2.2	250
1.9	3.5	2.2	250
1.9	4.0	2.2	250

## Data Availability

No new data were created or analyzed in this study. Data sharing is not applicable to this article.
